# Interim Estimate of Vaccine Effectiveness of BNT162b2 (Pfizer-BioNTech) Vaccine in Preventing SARS-CoV-2 Infection Among Adolescents Aged 12–17 Years — Arizona, July–December 2021

**DOI:** 10.15585/mmwr.mm705152a2

**Published:** 2021-12-31

**Authors:** Karen Lutrick, Patrick Rivers, Young M. Yoo, Lauren Grant, James Hollister, Krystal Jovel, Sana Khan, Ashley Lowe, Zoe Baccam, Hanna Hanson, Lauren E.W. Olsho, Ashley Fowlkes, Alberto J. Caban-Martinez, Cynthia Porter, Sarang Yoon, Jennifer Meece, Manjusha Gaglani, Joy Burns, Julie Mayo Lamberte, Flavia Nakayima Miiro, Adam Bissonnette, Lindsay LeClair, Preeta K. Kutty, James K. Romine, Elisha Stefanski, Laura J. Edwards, Katherine Ellingson, Joe K. Gerald, Edward J. Bedrick, Purnima Madhivanan, Karl Krupp, Lynn B. Gerald, Mark Thompson, Jefferey L. Burgess

**Affiliations:** ^1^Mel and Enid Zuckerman College of Public Health, University of Arizona, Tucson, Arizona; ^2^CDC COVID-19 Response Team; ^3^Abt Associates, Rockville, Maryland; ^4^Rocky Mountain Center for Occupational and Environmental Health, Department of Family and Preventive Medicine, University of Utah Health, Salt Lake City, Utah; ^5^Baylor Scott and White Health, Texas A&M University College of Medicine, Temple, Texas; ^6^Leonard M. Miller School of Medicine, University of Miami, Miami, Florida; ^7^Marshfield Clinic Research Institute, Marshfield, Wisconsin.

The BNT162b2 (Pfizer-BioNTech) mRNA COVID-19 vaccine has demonstrated high efficacy in preventing infection with SARS-CoV-2 (the virus that causes COVID-19) in randomized placebo-controlled Phase III trials in persons aged 12–17 years (referred to as adolescents in this report) (*1*); however, data on real-word vaccine effectiveness (VE) among adolescents are limited (*1*–*3*). As of December 2021, the Pfizer-BioNTech vaccine is approved by the Food and Drug Administration (FDA) for adolescents aged 16–17 years and under FDA emergency use authorization for those aged 12–15 years. In a prospective cohort in Arizona, 243 adolescents aged 12–17 years were tested for SARS-CoV-2 by reverse transcription–polymerase chain reaction (RT-PCR) each week, irrespective of symptoms, and upon onset of COVID-19–like illness during July 25–December 4, 2021; the SARS-CoV-2 B.1.617.2 (Delta) variant was the predominant strain during this study period. During the study, 190 adolescents contributed fully vaccinated person-time (≥14 days after receiving 2 doses of Pfizer-BioNTech vaccine), 30 contributed partially vaccinated person-time (receipt of 1 dose or receipt of 2 doses but with the second dose completed <14 days earlier), and 66 contributed unvaccinated person-time. Using the Cox proportional-hazards model, the estimated VE of full Pfizer-BioNTech vaccination for preventing SARS-CoV-2 infection was 92% (95% CI = 79%–97%), adjusted for sociodemographic characteristics, health information, frequency of social contact, mask use, location, and local virus circulation. These findings from a real-world setting indicate that 2 doses of Pfizer-BioNTech vaccine are highly effective in preventing SARS-CoV-2 infection among Arizona adolescents. CDC recommends COVID-19 vaccination for all eligible persons in the United States, including persons aged 12–17 years.*

The PROTECT^†^ study is a prospective cohort of persons aged 4 months–17 years initiated in Arizona in July 2021. The study seeks to understand the risk for COVID-19 and how well COVID-19 vaccines protect children and adolescents from SARS-CoV-2 infection and illness. PROTECT expanded to Florida, Texas, and Utah in late September 2021, and those sites will be included in future analyses. PROTECT is an ancillary study of the HEROES-RECOVER cohorts,^§^ which previously reported VE of COVID-19 vaccines among working adults aged 18–85 years using similar methods (*4*). PROTECT participants in Arizona were recruited from families of adults participating in the HEROES study and the general public. Upon enrollment, participants responded to electronic surveys collecting demographic, health and vaccination history, and prior SARS-CoV-2 infection information. Participants submitted self-collected (or parent-/guardian-collected) mid-turbinate nasal swabs weekly, irrespective of COVID-19–like illness symptoms, and collected an additional swab at the onset of any COVID-19–like illness. Self-reported signs and symptoms of COVID-19–like illness (fever, chills, cough, shortness of breath, sore throat, diarrhea, muscle or body aches, change in smell or taste, or loss of appetite or poor feeding) that occurred in the preceding 7 days were self-reported on the weekly nasal swab envelopes. Specimens were shipped on cold packs and tested by RT-PCR assay for SARS-CoV-2 at Marshfield Clinic Laboratory (Marshfield, Wisconsin). Receipt of COVID-19 vaccines was documented by self-report in electronic surveys and direct upload of vaccine card images by participants’ parents or guardians. The number of hours and percentage of time participants wore masks in school and in the community were also collected via self-reported electronic surveys upon enrollment and each subsequent month.

The primary outcome measure was time to RT-PCR–confirmed SARS-CoV-2 infection in vaccinated participants compared with that in unvaccinated participants. VE was calculated using the Anderson-Gill extension of the Cox proportional-hazards models, in which unvaccinated person-time included days before receiving the first dose of a COVID-19 vaccine, and fully vaccinated person-time included number of days following 14 days after receipt of the second of 2 Pfizer-BioNTech vaccine doses. For participants infected with SARS-CoV-2, the event date for this analysis was the earlier of the collection date of the first specimen to test positive or the symptom onset date. Unadjusted VE was calculated as 100% x (1 – hazard ratio for SARS-CoV-2 infection in vaccinated versus unvaccinated participants). An adjusted model used an inverse probability of treatment weighting approach (*5*) with individual propensities to be vaccinated during each week based on sociodemographic characteristics (age, sex, race/ethnicity, and household size); health information (chronic conditions and daily medication use); frequency of close social contact (school and community); percentage of time wearing masks (school and community); and local virus circulation (daily percentage of all SARS-CoV-2 tests performed in the local county returning a positive result). These predicted propensities were used to calculate stabilized weights, which were incorporated into a Cox proportional-hazards model. Robust SEs were used to account for the clustering of participants within the same household and correlation by stabilized weights. All analyses were conducted using SAS software (version 9.4; SAS Institute) or R software (version 4.1.2; R Foundation for Statistical Computing). This activity was approved by University of Arizona Institutional Review Board on which CDC relied. The study was conducted consistent with applicable federal law and CDC policy.^¶^

Among 1,478 participants aged 4 months–17 years in the full Arizona PROTECT cohort, 280 (18.9%) were aged 12–17 years; 32 (11.4%) of these participants were excluded based on a documented RT-PCR–positive SARS-CoV-2 test result before enrollment, three were excluded because of failure to complete weekly nasal swabs, one was excluded because vaccination information was incomplete, and one was excluded because the participant had received the mRNA-1273 (Moderna) COVID-19 vaccine, leaving 243 participants (86.8% of participants aged 12–17 years) in the analytic sample.

Approximately one half (51.4%) were male, 65.8% were from Tucson, most were aged 12–15 years (74.5%), White (87.7%), non-Hispanic (74.5%), and had private insurance (85.2%) (Table 1). Participants reported attending in-person school a mean of 28.2 (SE = 1.0) hours per week. They reported wearing a mask in school 73.3% (SE = 2.4) of the time; the SE, in part, reflects the variability in mask mandates across the state (*6*). Participants who received a positive SARS-CoV-2 test result during the study reported a lower percentage of time masked in school (48.6%, SE = 10.0) compared with those who did not receive a positive test result (75.7%, SE = 2.3) (p = 0.031). Participants also reported using masks in the community a mean of 58.5% (SE = 2.6) of the time overall, with participants who received positive SARS-CoV-2 test results reporting a lower mean percentage of community masked time (29.3%; SE = 9.0) compared with those who received negative test results (61.3%; SE = 2.7) (p = 0.003).

**TABLE 1:** Demographic and epidemiologic characteristics of adolescents aged 12–17 years in the Arizona PROTECT Pfizer-BioNTech COVID-19 vaccine effectiveness cohort (N = 243) — Arizona, July–December 2021

Characteristic	Participants	SARS-CoV-2 infection	No SARS-CoV-2 infection	p-value*	Unvaccinated	Vaccinated 1 dose	p-value*
	No. (col. %)	No. (row %)	No. (row %)		No. (row %)	No. (row %)	
**Total**	**243 (100.0)**	**21 (8.6)**	**222 (91.4)**	**—**	**49 (20.2)**	**194 (79.8)**	**—**
**Gender**							
Male	125 (51.4)	12 (9.6)	113 (90.4)	0.191	25 (20.0)	100 (80.0)	>0.999
Female	106 (43.6)	7 (6.6)	99 (93.4)		22 (20.8)	84 (79.2)	
Transgender	2 (0.8)	1 (50.0)	1 (50.0)		0 (0.0)	2 (100.0)	
None of these or did not respond	10 (4.1)	1 (10.0)	9 (90.0)		2 (20.0)	8 (80.0)	
**Site**							
Phoenix	49 (20.2)	5 (10.2)	44 (89.2)	0.003	10 (20.4)	39 (79.6)	0.594
Tucson	160 (65.8)	8 (5.0)	152 (95.0)		30 (18.8)	130 (81.2)	
Other	34 (14.0)	8 (23.5)	26 (76.5)		9 (26.5)	25 (73.5)	
**Age group, yrs**							
12–15	181 (74.5)	15 (8.3)	166 (91.7)	0.941	38 (21.0)	143 (79.0)	0.713
16–17	62 (25.5)	6 (9.7)	56 (90.3)		11 (17.7)	51 (82.3)	
**Ethnicity (all races)**							
Hispanic	64 (25.5)	3 (4.8)	59 (95.2)	0.298	10 (16.1)	54 (83.9)	0.383
Non-Hispanic	179 (74.5)	18 (9.9)	163 (90.1)		39 (22.7)	140 (77.2)	
**Race (all ethnicities)**							
White	216 (87.7)	20 (9.4)	196 (90.6)	0.484	40 (18.8)	175 (81.2)	0.041
Other races^†^	27 (12.3)	1 (3.3)	26 (96.7)		9 (36.7)	19 (63.3)	
**Household composition**							
1 child per household	58 (23.9)	3 (5.2)	55 (94.8)	0.422	10 (17.2)	48 (82.8)	0.654
≥2 children per household	185 (76.1)	18 (9.7)	167 (90.3)		39 (21.1)	146 (78.9)	
**Swab adherence^§^**							
>80%	194 (79.8)	19 (9.8)	175 (90.2)	0.264	35 (18.0)	159 (82.0)	0.149
**Chronic conditions^¶^**							
≥1	25 (10.3)	4 (16.0)	21 (84.0)	0.247	7 (28.0)	18 (72.0)	0.443
None	218 (89.7)	17 (7.8)	201 (92.2)		42 (19.3)	176 (80.7)	
**Insurance**							
Private	207 (85.2)	17 (8.2)	190 (91.8)	0.387	39 (18.8)	168 (81.2)	0.409
None or did not respond	18 (7.4)	3 (16.7)	15 (83.3)		5 (27.8)	13 (72.2)	
Public	18 (7.4)	1 (5.6)	17 (94.4)		5 (27.8)	13 (72.2)	
**Potential virus exposure and mask use (hours weekly), no. (col. %)**							
Hours attending school, mean (SE)	28.2 (1.0)	22.2 (3.8)	28.8 (1.0)	0.178	25.2 (2.4)	29.0 (1.0)	0.308
% time masked, school, mean (SE)	73.3 (2.4)	48.6 (10.0)	75.7 (2.3)	0.031	61.6 (6.1)	76.2 (2.5)	0.216
Hours in community, mean (SE)	10.4 (0.8)	11.9 (2.1)	10.3 (0.9)	0.174	11.6 (1.7)	10.1 (1.0)	0.228
% time masked, community, mean (SE)	58.5 (2.6)	29.3 (9.0)	61.3 (2.7)	0.003	39.5 (6.2)	63.2 (2.8)	0.002

**Abbreviations: **Col = column; PROTECT = Pediatric Research Observing Trends and Exposures in COVID-19 Timelines.

* P-values comparing the percentage of persons with SARS-CoV-2 infections to those not infected by sociodemographic and health categories and comparing the percentage of vaccinated persons to those not vaccinated by these categories, calculated using Pearson’s chi-square test (cells with ≥5 observations) or Fisher’s exact test (cells with <5 observations). P-values for continuous variables calculated using the Mann-Whitney test.

^†^ All participants in the “Other races” category were collapsed into a single group because of small numbers.

^§^ Number and percentage of participants who completed weekly nasal swab throughout the analysis period.

^¶^ Chronic conditions included asthma or chronic lung disease, cancer, diabetes, heart disease, hypertension, immunosuppression or autoimmune disorder, kidney disease, liver disease, neurologic or neuromuscular disorder, or other chronic conditions.

During the study period, 66 participants contributed 4,288 unvaccinated person-days, 30 contributed 909 partially vaccinated person-days, and 190 contributed 21,693 fully vaccinated person-days (Table 2). Most (n = 171, 70.3%) vaccinated participants entered the study fully vaccinated. The median number of fully vaccinated person-days during the analysis period was 119 (IQR = 105–133 days).

**TABLE 2:** Contributing participants, total person-days, number of RT-PCR–confirmed SARS-CoV-2 infections by vaccination status, and estimated Pfizer-BioNTech COVID-19 vaccine effectiveness for full vaccination in preventing infection among vaccine-eligible adolescents aged 12–17 years (N = 243) — Arizona, July–December 2021

Pfizer COVID-19 vaccination status	No. of contributing participants*	Total person-days	No. of days, median (IQR)	No. of SARS-CoV-2 infections	VE, % (95% CI)	
					Unadjusted	Adjusted^†,§^
**Unvaccinated**	66	4,288	62 (23–98)	16	—	—
**Partially vaccinated (≥14 days after dose 1 to day 13 after dose 2)**	30	909	21 (20–28)	0	—	—
**Fully vaccinated (≥14 days after dose 2)**	190	21,693	119 (105–133)	5	94 (83–98)	92 (79–97)

**Abbreviations:** RT-PCR = reverse transcription–polymerase chain reaction; SMD = standardized mean difference; VE = vaccine effectiveness.

* Contributing participants in vaccination categories did not equal the number of participants in the study because participants could contribute to more than one vaccination category since vaccination status varies by time.

^†^ Adjusted VE is inversely weighted for propensity to be vaccinated; all covariates met balance criteria of SMD<0.2 after weighting except community mask use and local virus circulation (SMD = 0.228 and 0.288, respectively), but community mask use was only found to change VE estimate by ≥5% when added to the model and was therefore included as a covariate in the Cox regression model for VE.

^§^ Five participants missing community mask use were excluded from analysis; this exclusion did not affect the VE estimate.

Twenty-one persons (8.6%) received positive RT-PCR SARS-CoV-2 test results (Table 1). RT-PCR–confirmed infection was more prevalent among residents of areas other than Tucson or Phoenix (p = 0.003). The majority (n = 18, 85.7%) of participants with RT-PCR–confirmed infection reported COVID-19–like illness. The remaining three participants reported being asymptomatic. Two participants with RT-PCR–confirmed infections, both unvaccinated and from the same household, sought outpatient medical care for their illness.

During the 4,288 unvaccinated person-days, 16 RT-PCR–confirmed infections were identified (incidence rate = 3.73 per 1,000 person-days) (Table 2). During the 909 person-days <14 days after receipt of the second vaccine dose, when persons were considered partially vaccinated, no RT-PCR–confirmed infections were identified. Five RT-PCR–confirmed infections occurred during 21,693 fully vaccinated person-days (incidence rate = 0.23 per 1,000 person-days). Estimated unadjusted VE of full vaccination for preventing SARS-CoV-2 infection was 94% (95% CI = 83%–98%). Estimated adjusted VE of full vaccination for preventing SARS-CoV-2 infection was 92% (95% CI = 79%–97%).

## Discussion

Analysis of a prospective Arizona cohort of adolescents found adjusted VE for full vaccination with 2 doses of Pfizer-BioNTech vaccine to be 92% against RT-PCR–confirmed SARS-CoV-2 infection, indicating that the Pfizer-BioNTech COVID-19 vaccine is highly effective in real-world conditions among adolescents aged 12–17 years.

These findings are consistent with those from previous Phase III trials (*1*,*7*) and recent observational studies of mRNA VE against severe COVID-19 in adolescents and young adults (*3*,*8*). The scientific rigor of these findings is enhanced by the study’s prospective design and the participants’ weekly specimen collections. The observation period for this analysis coincided with the period of Delta variant predominance in the United States and with return to in-person K–12 instruction in Arizona schools, with potentially higher rates of exposure.

The findings in this report are subject to at least five limitations. First, VE point estimates should be interpreted with caution given the moderately wide CIs, attributable in part to the limited number of unvaccinated person-days relative to fully vaccinated person-days, and a small overall sample size. Second, although several potential confounders were controlled for, including differences in mask use between vaccinated and unvaccinated participants, residual and unmeasured confounding might have occurred. Third, self-collection of specimens and use of nasal rather than nasopharyngeal swabs could reduce sensitivity of virus detection by RT-PCR but might have increased participation, because studies indicate that nasal swabs are more acceptable to participants (*9*); if the difference in sensitivity of virus detection between the two methods disproportionately affected those who received the vaccine, VE would be overestimated. Fourth, if vaccination attenuates viral RNA shedding among children, as has been noted in some studies of adults (*4*), this effect would also result in overestimation of VE by reducing RT-PCR detection among infected but vaccinated participants. Finally, the study might not be generalizable to other populations. The study was restricted to adolescents in Arizona and might not be representative of the racial or ethnic distribution in Arizona nor the United States. In addition, participants reported very few chronic conditions and low rates of obesity; previous studies have indicated that VE has not varied by chronic conditions except for among immunocompromised adults (*10*). The study was also restricted to persons aged 12–17 years; it is not known whether these findings can be generalized to children aged 5–11 years, who are now eligible to receive the Pfizer-BioNTech vaccine under an emergency use authorization.

The VE estimates described in this report for the Pfizer-BioNTech vaccine in real-world conditions during the period of Delta variant predominance corroborate and expand upon the VE estimates from other recent studies in adolescents (*1*,*7*) and reinforce previous findings that current vaccination efforts are resulting in substantial preventive benefits among adolescents aged 12–17 years. CDC recommends COVID-19 vaccination for all eligible persons in the United States, including adolescents aged 12–17 years.

**Summary**

**What is already known about this topic?**

The Pfizer-BioNTech COVID-19 vaccine has been shown to be effective in preventing SARS-CoV-2 infection in adolescents in randomized placebo-controlled Phase III trials.

**What is added by this report?**

A prospective cohort of 243 adolescents aged 12–17 years in Arizona completed weekly SARS-CoV-2 testing by nasal swab for 19 consecutive weeks. Under real-world conditions, vaccine effectiveness of full immunization (completion of the second in a 2-dose series ≥14 days earlier) was 92% against SARS-CoV-2 infections irrespective of symptom status.

**What are the implications for public health practice?**

In real-world conditions among adolescents aged 12–17 years, the Pfizer-BioNTech vaccine was highly effective in preventing SARS-CoV-2 infection.

## Footnotes

*
https://www.cdc.gov/coronavirus/2019-ncov/vaccines/recommendations/children-teens.html

† Pediatric Research Observing Trends and Exposures in COVID-19 Timelines (PROTECT).

§ Arizona Healthcare, Emergency Response and Other Essential Workers Surveillance Study (HEROES) and Research on the Epidemiology of SARS-CoV-2 in Essential Response Personnel (RECOVER) cohorts.

¶ 45 C.F.R. part 46; 21 C.F.R. part 56; 42 U.S.C. Sect. 241(d), 5 U.S.C. Sect. 552a, 44 U.S.C. Sect. 3501 et seq.
